# Temporal Expectations Prepare Visual Working Memory for Behavior

**DOI:** 10.1162/jocn_a_01626

**Published:** 2020-12-01

**Authors:** Wen Jin, Anna C. Nobre, Freek van Ede

**Affiliations:** 1 Oxford Centre for Human Brain Activity, Wellcome Centre for Integrative Neuroimaging; 2 University of Oxford; 3 Oxford Centre for Human Brain Activity, Wellcome Centre for Integrative Neuroimaging; 4 University of Oxford; 5 Oxford Centre for Human Brain Activity, Wellcome Centre for Integrative Neuroimaging; 63 Institute for Brain and Behavior Amsterdam, Vrije Universiteit Amsterdam

## Abstract

Working memory enables us to retain past sensations in service of anticipated task demands. How we prepare for anticipated task demands during working memory retention remains poorly understood. Here, we focused on the role of time—asking how temporal expectations help prepare for ensuing memory-guided behavior. We manipulated the expected probe time in a delayed change-detection task and report that temporal expectation can have a profound influence on memory-guided behavioral performance. EEG measurements corroborated the utilization of temporal expectations: demonstrating the involvement of a classic EEG signature of temporal expectation—the contingent negative variation—in the context of working memory. We also report the influence of temporal expectations on 2 EEG signatures associated with visual working memory—the lateralization of 8- to 12-Hz alpha activity, and the contralateral delay activity. We observed a dissociation between these signatures, whereby alpha lateralization (but not the contralateral delay activity) adapted to the time of expected memory utilization. These data show how temporal expectations prepare visual working memory for behavior and shed new light on the electrophysiological markers of both temporal expectation and working memory.

## INTRODUCTION

Working memory (Baddeley, 1992) enables us to retain past sensations with the purpose of guiding adaptive future behavior. In considering this *purpose-oriented* nature of working memory (de Vries, Slagter, & Olivers, 2020; van Ede, 2020; Nobre & Stokes, 2019; van Ede, Chekroud, Stokes, & Nobre, 2019; Myers, Stokes, & Nobre, 2017; Chatham & Badre, 2015; D'Esposito & Postle, 2015; Carlisle, Arita, Pardo, & Woodman, 2011; Rainer, Rao, & Miller, 1999; Chelazzi, Miller, Duncan, & Desimone, 1993), it is relevant to consider not only *what* we encode and retain in working memory (Lee, Kravitz, & Baker, 2013; Serences, Ester, Vogel, & Awh, 2009; McNab & Klingberg, 2008; Vogel, McCollough, & Machizawa, 2005; Griffin & Nobre, 2003) but also *when* we expect to utilize the contents in working memory in service of ensuing behavior.

Despite a growing realization of the important role of temporal expectations in guiding behavior (Nobre & van Ede, 2018), most studies to date have investigated temporal expectations in service of relatively pure sensory (Rohenkohl, Cravo, Wyart, & Nobre, 2012; Vangkilde, Coull, & Bundesen, 2012; Jaramillo & Zador, 2011; Lakatos, Karmos, Mehta, Ulbert, & Schroeder, 2008; Lange & Röder, 2006; Coull & Nobre, 1998; Jones & Boltz, 1989) or motor (Heideman, Quinn, Woolrich, van Ede, & Nobre, 2020; Heideman, van Ede, & Nobre, 2018; Los, Kruijne, & Meeter, 2017; van Elswijk, Kleine, Overeem, & Stegeman, 2007; Praamstra, 2006; Janssen & Shadlen, 2005; Schoffelen, Oostenveld, & Fries, 2005) tasks. In everyday situations, however, we often rely on detailed contents of working memory to inform and guide behavior (van Ede et al., 2019; Myers et al., 2017; Chatham & Badre, 2015; Olivers, Peters, Houtkamp, & Roelfsema, 2011; Chelazzi et al., 1993). Yet, less is understood about the ways in which temporal expectations are utilized while we anticipate using detailed information in working memory to guide our behavior.

Complementing other recent studies on the role of temporal expectations in working memory (Boettcher, Gresch, Nobre, & van Ede, 2020; Zokaei, Board, Manohar, & Nobre, 2019; Wilsch, Henry, Herrmann, Herrmann, & Obleser, 2018; Olmos-Solis, van Loon, Los, & Olivers, 2017; van Ede, Niklaus, & Nobre, 2017; Wilsch, Henry, Herrmann, Maess, & Obleser, 2015), we sought to investigate the behavioral consequences and neural signatures of temporal expectation in the context of a well-studied change-detection task of visual working memory, with lateralized encoding displays (Vogel et al., 2005; Vogel & Machizawa, 2004). We investigated the consequences of temporal expectations on memory-guided behavior in this task, by manipulating the time at which the contents of working memory would likely be probed.

EEG measurements enabled us to address additional relevant questions regarding key electrophysiological signatures of temporal expectations and of visual working memory retention after lateralized displays. These signatures involved the contingent negative variation or CNV (Boettcher, Stokes, Nobre, & van Ede, 2020; Cravo, Rohenkohl, Santos, & Nobre, 2017; Praamstra, Kourtis, Kwok, & Oostenveld, 2006; Miniussi, Wilding, Coull, & Nobre, 1999; Weinberg, 1972; Walter, Cooper, Aldridge, McCallum, & Winter, 1964), the lateralization of 8- to 12-Hz alpha activity (Hakim, Adam, Gunseli, Awh, & Vogel, 2019; van Ede, 2018; Myers, Walther, Wallis, Stokes, & Nobre, 2015; Wallis, Stokes, Cousijn, Woolrich, & Nobre, 2015; Lozano-Soldevilla, ter Huurne, Cools, & Jensen, 2014; van Dijk, van der Werf, Mazaheri, Medendorp, & Jensen, 2010; Sauseng et al., 2009), and the contralateral delay activity or CDA (Hakim et al., 2019; Schmidt & Zelinsky, 2017; Luria, Balaban, Awh, & Vogel, 2016; Kuo, Stokes, & Nobre, 2012; Carlisle et al., 2011; van Dijk et al., 2010; Vogel et al., 2005; Vogel & Machizawa, 2004). Specifically, we addressed whether the CNV also supports temporal expectation during working memory and whether alpha lateralization and the CDA—two spatially selective markers of attention and/or retention in visual working memory—are each sensitive to temporal expectation and adapt to the time of expected memory utilization.

We hypothesized that if temporal expectations are utilized to prepare for upcoming visual-working-memory-guided behavior, then performance to early probes should be better when probes were expected to appear early as opposed to late. We further hypothesized that the utilization of temporal expectations should be traceable in the CNV, yielding a more negative frontal potential ahead of the early probe when the probe is expected early as opposed to late. Finally, we reasoned that if working memory retention is modulated by temporal expectation, this would be reflected in its related neural markers. Accordingly, we tested for modulations of alpha lateralization and the CDA to test whether they tracked modulation in working memory retention. In other words, we investigated whether these signatures depend not only on *what* information is retained in working memory (left vs. right items) but also on *when* this information is expected to become required for guiding behavior.

## METHODS

### Participants

Twenty-five healthy human volunteers (7 men, mean age = 26, range: of 18–35) participated in the study. Sample size was set based on prior studies from the laboratory with similar outcome variables (van Ede et al., 2017, 2019). All participants had normal or corrected-to-normal vision. Data from one participant had to be excluded from the analysis because of below-chance task performance (49.8% correct responses, compared with *M* = 76.02, *SE* = 1.96% in the remaining sample). All participants provided written informed consent before participation and were paid £15 per hour. The study was approved by the central university research ethics committee of the University of Oxford.

### Task and Procedure

**Figure 1.**
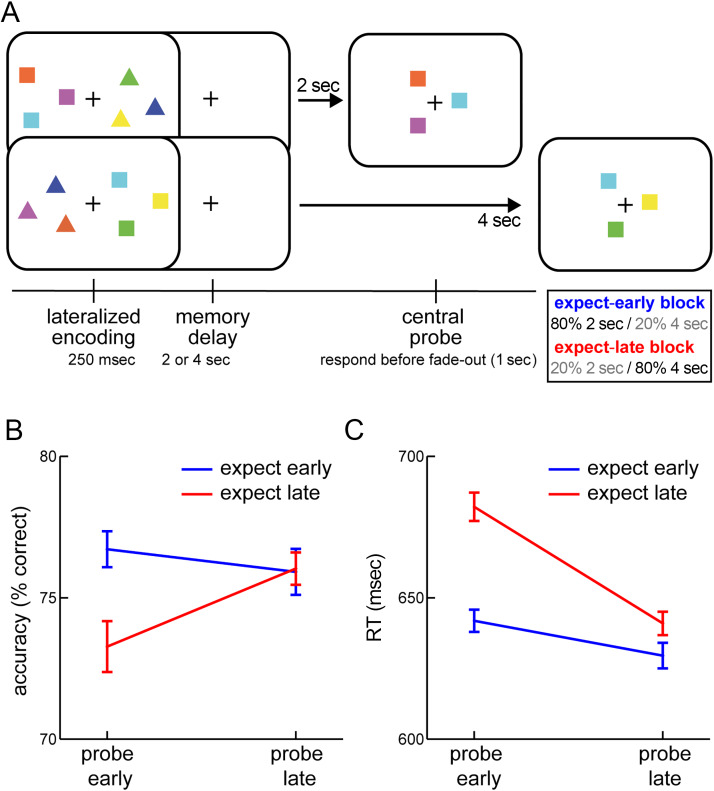
Temporal expectations shape working-memory-guided behavior. (A) Visual working memory task with a standard temporal expectation manipulation. Participants were instructed to selectively remember the squares or the triangles. After a memory delay of 2 or 4 sec, a central probe was presented and participants judged whether the probe configuration remained the same (4-sec example) or changed (2-sec example) from the encoded configuration of the relevant shapes. Across blocks, probes were either most likely (80%) to occur early (2 sec) or late (4 sec), but would occur at the unexpected time in 20% of the trials. (B) Percentage correct responses as a function of expected probe time and actual memory delay. (C) RT as a function of expected probe time and actual memory delay. Error bars indicate ± 1 *SEM* calculated across participants (*n* = 24), after within-subject correction (Cousineau, 2005).

Participants performed a delayed visual change-detection working memory task with lateralized encoding displays, in which we manipulated temporal expectations regarding expected probe time across blocks (Figure 1A).

Participants sat approximately 90 cm in front of a computer monitor (100-Hz refresh rate). At memory encoding, one side of the display always contained three colored squares, whereas the other side contained three colored triangles. Each shape had a unique color from a pool of six possible colors (red, green, blue values: [255, 107, 64], [255, 255, 64], [64, 255, 107], [64, 64, 255], [64, 255, 255], and [255, 64, 255]). Individual shapes were approximately 1° visual angle in width and height and were positioned at a random point along the outer edge of an invisible circle (6.4° diameter; anchored at 6.4° to the left and right of fixation). Before each of two experimental sessions, participants were explicitly instructed to remember one set of shapes (say squares) while ignoring the other set (triangles). Which set of shapes was relevant was counterbalanced across sessions. Irrelevant shapes served to balance the visual encoding display (and to require item selection at encoding), such that any lateralized patterns of neural activity could be attributed to the selection and retention of the relevant memory items (rather than bottom–up visual responses to the display). The positioning of the relevant and irrelevant shapes to the left and right locations on the screen was randomly varied across trials.

Encoding displays containing the 2 × 3 shapes were presented for 250 msec, followed by a memory delay period of either 2 or 4 sec in which only the fixation cross (2° in width and height) remained on the screen. After the memory delay, a probe display was presented that always contained the three relevant shapes, but of which two of the shapes (from the relevant shape set) had either swapped colors (change) or not (no change). Change and no-change trials were equally likely and randomly intermixed. Probe displays were lpresented centrally, but otherwise preserved the spatial configuration of the relevant shape set. Participants responded using the keyboard in front of them and pressed “M” (for “match”) if they thought the color configuration had remained the same, or “C” (for “change”) if they thought it had changed.

To encourage holding the memory items ready for guiding behavior, we instructed participants to make their change/no-change report within 1 sec after probe onset. To this end, we gradually faded the visibility of the probe by adjusting its transparency levels from 0–100% over a 1-sec period, starting at probe onset. Participants were explicitly told that responses that would be received after the probe had disappeared would be counted as incorrect.

The program provided feedback on performance as soon as a response was collected or the maximum response time had been reached. The fixation cross changed (for 300 msec) to green if a correct response was made, or to red otherwise. The next trial began after an intertrial interval between 750 and 1000 msec.

The critical manipulation in the current study involved the expected probe time. In “expect-early” blocks, 80% of trials (32/40 per block) had a memory delay of 2 sec, whereas 20% of trials (8/40 per block) had a delay of 4 sec. In contrast, in “expect-late” blocks, 80% of trials had a memory delay of 4 sec, whereas only 20% of trials had a delay of 2 sec. Expect-early and expect-late blocks were randomly interleaved. Participants were explicitly informed what block type they were in before the block started, as signaled by a preblock instruction screen.

Participants completed two consecutive sessions of the task of approximately 45 min each, with a 10- to 15-min break in between them. The two sessions each contained 10 blocks (5 per block type) of 40 trials, yielding 800 trials per participant.

### Visual Localizer

In between each block of the main working memory task, we included visual localizer modules that served to find the posterior electrodes that best captured visual processing of the stimuli that we used in the main task. In each localizer display, we presented a single set of three colored shapes (three squares or three triangles), either to the left or to the right of fixation. Participants were instructed to maintain fixation on the central cross, without any further task. Individual localizer displays were presented for 250 msec, followed by an interstimulus interval of 400–600 msec. Each localizer module contained 80 stimulus displays, yielding 1,600 localizer displays across the two sessions (800 per side).

The rationale for including localizers was motivated by the use of similar localizers in several of our prior studies (e.g., van Ede et al., 2017; van Ede, Jensen, & Maris, 2010). Localizers provided an independent means to find left and right electrodes that were maximally sensitive to visual processing of the contralateral (vs. ipsilateral) visual field—and to do so on a participant-by-participant basis. Having found these participant-specific electrodes that were maximally sensitive to visual processing of the contralateral visual field, we were then able to use these electrodes to focus our analysis on lateralized signatures of visual processing associated with working memory retention—alpha lateralization and the CDA. By focusing on these independently selected electrodes, we were thus able to increase the sensitivity of our analysis. Because the localizer-based electrode selection served only as a “proxy” for the set of relevant posterior electrodes (there was no strict guarantee that these lateralized working memory signatures would occur in precisely the same electrodes), we additionally present the topographies of these lateralized EEG modulations.

### Analysis of Behavioral Data

We considered two measures of performance: accuracy and RT. For accuracy, we calculated the percentage of trials with a correct response. Incorrect response trials included trials in which the wrong button was pressed or in which no button press was recorded in the 1-sec response window during which the probe gradually faded. Participants were explicitly told that responses occurring after the probe had disappeared would be counted as incorrect (and were given negative feedback immediately after the end of the response window was reached). For RTs, we only considered trials in which a response was collected (95.84 ± 1.22% of trials).

To quantify the effects of temporal expectations on working-memory-guided performance, we used a 2 × 2 repeated-measures ANOVA with the factors Block Type (expect early/late) and Memory Delay (probe early/late) and used follow-up paired-samples *t* tests to test for temporal expectations effects at each memory delay separately. For this, we directly compared expect-early and expect-late conditions, separately for performance after early and late probes. For these post hoc *t* tests, we additionally report Bonferroni-corrected *p* values that we denote as “*p*_Bonferroni_.”

### EEG Acquisition

EEG was acquired using Synamps amplifiers and Neuroscan acquisition software. We used 61 electrodes that we placed according to the international 10–10 positioning system (Chatrian, Lettich, & Nelson, 1985). During acquisition, the left mastoid served as the reference. A right mastoid measurement was included for off-line referencing to the average of both mastoids. We positioned the ground electrode on the left upper arm. Vertical and horizontal EOG were measured using electrodes placed to the lateral side of each eye (horizontal EOG), as well as above and below the left eye (vertical EOG). Data were acquired with a hardware filter between 0.1 and 200 Hz, digitized at 1000 Hz, and stored for off-line analysis.

### EEG Analysis

Data were analyzed in using the FieldTrip toolbox (Oostenveld, Fries, Maris, & Schoffelen, 2011) in MATLAB (The MathWorks). We used independent component analysis to correct for ocular contributions to the EEG. After independent component analysis correction, we discarded trials with exceptionally high variance on the basis of visual inspection (using the function *ft_rejectvisual* with the summary method). On average, 686 ± 15 (out of 800) trials were retained for analysis per participant.

We focused on two complementary sets of analyses—focusing on a global signature of temporal expectation (the CNV) and on the spatially specific memory-retention signatures that lateralized according to the side of the memory items (alpha lateralization and the CDA).

#### Analysis of CNV

Data were baseline corrected by subtracting the average potential in the 250-msec window preceding encoding onset, and averaged across trials. We used a predefined cluster of frontal EEG electrodes, centered on electrode Fz together with its immediate neighbors: (FCz, AFz, Fz, F1, F2), as in the study of Boettcher, Stokes, et al. (2020). Data were averaged across the selected electrodes and compared between trials in which the probe was expected to occur early (expect-early blocks) or late (expect-late blocks). To increase visualization of this slow potential, we smoothed the CNV time courses with a Gaussian kernel with an *SD* of 25 msec.

#### Analysis of Lateralized Alpha and CDA

To increase sensitivity for the analysis of neural lateralization, we applied a surface-Laplacian transform (Perrin, Pernier, Bertrand, & Echallier, 1989) that increases separability of nearby sources of activity (here, activity in left and right visual electrodes; note that we deliberately did not apply the same transform to the more global CNV analysis, as the CNV is relatively widespread).

We used clusters of left and right posterior electrodes that were selected on a participant-by-participant basis, based on the data from our independent visual localizer modules (see Visual Localizer section)—as in our prior study (van Ede et al., 2017). In short, we compared responses to localizer displays with left versus right items, and compared 8- to 12-Hz alpha activity in the 150- to 400-msec poststimulus window. Our choice to focus on the 150- to 400-msec window was based on our prior experience with similar localizers (e.g., van Ede et al., 2010, 2017), which have indicated this as the approximate window in which the induced alpha response to a sensory stimulus is most pronounced. By starting 150 msec after stimulus onset, we minimize the contribution of the initial evoked response, which leads to an increase in power across a wide range of frequencies, thereby obscuring any stimulus-induced alpha decreases in the first 150 msec after stimulus onset. We then selected the posterior electrodes that showed the clearest difference between left and right localizer stimuli based on visual inspection. We selected electrodes from among the following potential posterior electrodes: left: (P7, P5, P3, P1, PO7, PO3, O1); right: (P8, P6, P4, P2, PO8, PO4, O2). We selected no less than four and no more than six electrodes per side. The number of participants (out of 24) for which a given electrode was included were P7 (21), P5 (24), P3 (21), P1 (2), PO7 (24), PO3 (23), O1 (11), P8 (18), P6 (23), P4 (22), P2 (4), PO8 (24), PO4 (24), and O2 (15).

For the analysis of the CDA, like for the CNV, we applied a baseline correction using a 250-msec pre-encoding baseline and smoothed the time courses with a Gaussian kernel with an *SD* of 25 msec. We considered the data in the selected left and right posterior electrode clusters, as a function of whether the to-be-memorized shapes had been presented to the left or right of fixation at encoding. Per electrode cluster, the CDA was calculated by subtracting conditions where the memorized items had been contralateral versus ipsilateral to the electrode cluster under investigation. CDA waveforms were subsequently averaged between the left and right electrode clusters to yield a single CDA waveform per temporal expectation condition.

For the analysis of alpha lateralization, we first applied a time–frequency analysis using a short-time Fourier transform of Hanning-tapered data, as implemented in FieldTrip (Oostenveld et al., 2011). We estimated spectral power between 5 and 25 Hz (in steps of 1 Hz) and used a 300-msec sliding time window that we advanced over the data in steps of 30 msec. As for the CDA, time–frequency maps of power were contrasted between trials in which the memory items were contralateral versus ipsilateral to each electrode cluster. For normalization purposes, we expressed this difference in spectral power as a percentage change: ((contra − ipsi) / (contra + ipsi)) × 100. To visualize the time courses of alpha lateralization in expect-early and expect-late conditions, we extracted the activity in the predefined 8- to 12-Hz alpha band.

Topographical maps of alpha lateralization and CDA were obtained by applying the same procedures to all symmetrical electrode pairs and plotting the results in the right electrode of each pair.

#### Avoiding Neural Contamination by Early Probes

Our EEG analyses focused on the early memory delay period where temporal expectation effects are known to be most pronounced (e.g., Heideman, Rohenkohl, et al., 2018; Nobre & van Ede, 2018; Nobre, 2001). This allowed us to compare neural activity between all trials in expect-early and expect-late blocks (regardless of whether the probe actually appeared after 2 or 4 sec). To avoid contamination by any probe-related activity into the presented analysis—which would be problematic given the larger number of early probes in the expect-early condition—we only considered data for which we could be sure that there was no contamination by the probe (as in van Ede et al., 2017). For the CNV and CDA analyses, we substituted the data from all trials with a 2-sec memory delay with “Not a Number” from the time of probe onset (2250 msec after encoding onset). For the time–frequency analysis, we did the same, but started filling the contaminated data with Not a Number 150 msec earlier, to deal with the fact that we had used a 300-msec window sliding time window (yielding 150-msec smearing to each side).

#### Statistical Approach to EEG Data

We compared expect-early and expect-late conditions using cluster-based permutation analyses (Maris & Oostenveld, 2007), as implemented in the FieldTrip toolbox (Oostenveld et al., 2011). We used the default clustering settings, with 10,000 permutations. We applied these analyses on the data extracted from the predefined electrode clusters. All topographical analyses served only to confirm the physiological plausibility (van Ede & Maris, 2016) of the identified patterns and were not subjected to further inferential statistical tests.

## RESULTS

Participants performed a visual working memory task in which we manipulated the time at which information in working memory would become relevant for guiding behavior (Figure 1A). Participants were instructed to remember the squares or the triangles and to judge whether the configuration of these relevant shapes had changed or not between the encoding display and the probe display. In expect-early blocks, 80% of trials had a memory delay of 2 sec, whereas 20% of trials had a delay of 4 sec. In contrast, in expect-late blocks, 80% of trials had a memory delay of 4 sec, whereas only 20% of trials had a delay of 2 sec.

### Temporal Expectations Are Utilized during Visual Working Memory: Behavior and CNV

To assess whether participants utilized the temporal predictability of the probe array to facilitate their working-memory-guided behavior, we investigated behavioral accuracy and RTs when the probe array occurred early or late (memory delay) as a function of whether it was expected to occur early or late (block type).

For accuracy (Figure 1B) we observed a significant interaction between the time of probing (Memory Delay) and the expected probe time (Block Type), *F*(1, 23) = 4.329, *p* = .049, η_p_^2^ = .158), without a main effect of Memory Delay (*p* = .202) or Block Type (*p* = .095). When visual working memory was probed early, participants were substantially better when they also expected the probe to occur early as opposed to when they expected the probe to occur late (*M* = 76.7 vs. 73.3% correct; *t*(23) = 2.522, *p* = .019, *p*_Bonferroni_ = 0.038, *d* = 0.515). In contrast, when memory was probed late, there was no longer an effect of temporal expectation (*M* = 75.9 vs. 76.0% correct; *p* = .926). This lack of effect after the long memory delay is in line with ample prior studies on temporal expectation in the domains of perception and action (Nobre & van Ede, 2018 for review) and is likely attributed to the fact that, once the short delay has passed, participants can update their expectations according to the equated temporal conditional probability at the later interval.

Importantly, this pattern of data shows that the worse performance when the probe unexpectedly comes early cannot be because of a weaker memory trace per se because performance in these expect-late blocks recovers when probed at the late interval (despite the fact that now more time has passed since encoding). Instead, this is likely attributed to a suboptimal readiness (or “coding format”) of the memory array, when prompted to perform the working memory task earlier than expected (see also van Ede et al., 2017).

For RT (Figure 1C), we observed a qualitatively similar pattern. We found a significant interaction between Memory Delay and Block Type, *F*(1, 23) = 15.9324, *p* = 5.745e−4, η_p_^2^ = .409) that was again constituted by a particularly robust benefit of matching expectation for early probes (*M* = 630.4 vs. 666.0 ms; *t*(23) = −6.271, *p* = 2.126e−6, *p*_Bonferroni_ = 4.253e−6, *d* = −1.280), although a trend for faster responses in expect-early blocks persisted after late probes (*M* = 620.1 vs. 628.7 ms; *t*(23) = −2.087, *p* = .048, *p*_Bonferroni_ = 0.096, *d* = −0.426). RTs additionally showed a main effect of Memory Delay, *F*(1, 23) = 15.461, *p* = 6.659e−4, η_p_^2^ = .683) and Block Type, *F*(1, 23) = 37.064, *p* = 3.287e−6, η_p_^2^ = .650), with reactions being generally faster after late probes and in expect-early blocks.

**Figure 2.**
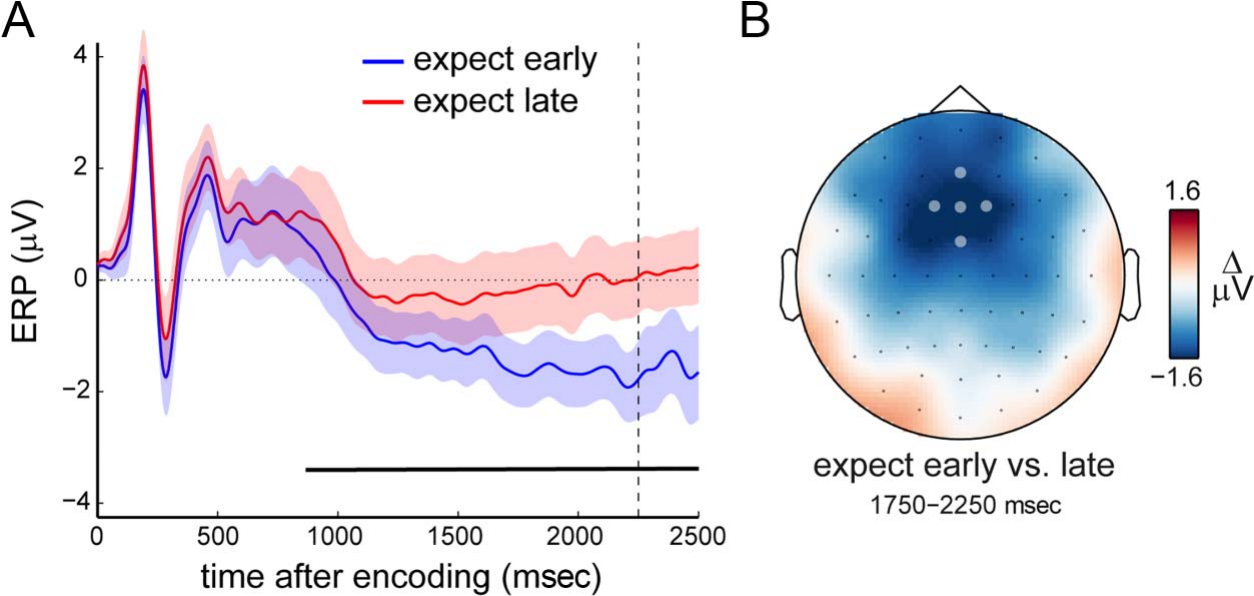
A CNV potential associated with temporal expectations during working memory. (A) Event-related potentials in predefined frontal electrodes (marked in B; [FCz, AFz, Fz, F1, F2]; as in Boettcher, Stokes, et al., 2020), as a function of expected probe time. Dashed vertical line indicates the time at which an early probe could occur. Black horizontal line indicates significant difference between conditions after a cluster-based permutation analysis (Maris & Oostenveld, 2007). Shaded areas indicate ± 1 *SEM*, calculated across participants (*n* = 24). (B) Topography associated with the potential difference shown in A.

EEG measurements provided complementary evidence that participants prepared for the time of expected memory utilization while engaging in visual working memory retention. Focusing on the initial memory interval—in which expect-early and expect-late blocks are known to differ most (Nobre & van Ede, 2018; Nobre, 2001)—we observed the gradual emergence of a frontal scalp potential that was more negative when memory was expected to be probed early versus late (Figure 2A; black line; cluster *p* < .0001). This potential likely reflects the CNV (Boettcher, Stokes, et al., 2020; Cravo et al., 2017; Praamstra et al., 2006; Miniussi et al., 1999; Weinberg, 1972; Walter et al., 1964), as also evidenced by its clear frontal topography (Figure 2B).

Together, these behavioral and EEG data each make clear that participants take the expected time of prospective memory use into account while engaging in visual working memory. They show that temporal expectations are utilized to benefit performance at the time of expected memory use and that such expectations are associated with a CNV during visual working memory retention.

Notably, in other work from our laboratory, we recently showed how the CNV can be sensitive to the contents of working memory, even when carefully controlling response demands between conditions (Boettcher, Stokes, et al., 2020). It thus remains a theoretical possibility that the CNV modulation in the current study too may be related to differences in working memory processes between temporal expectation conditions, in addition to changes in response readiness.

Owing to the lateralized nature of our displays, we were also in the position to investigate patterns of lateralized neural activity according to the encoded location of the relevant memory items. We focused on two human EEG signatures commonly associated with visual working memory after such displays: the lateralization of posterior 8- to 12-Hz alpha activity (Hakim et al., 2019; van Ede, 2018; Myers, Walther, et al., 2015; Lozano-Soldevilla et al., 2014; van Dijk et al., 2010; Sauseng et al., 2009) and the CDA (Hakim et al., 2019; Schmidt & Zelinsky, 2017; Luria et al., 2016; Carlisle et al., 2011; van Dijk et al., 2010; Vogel et al., 2005; Vogel & Machizawa, 2004).

### Timed Reorganization of Alpha Lateralization in Anticipation of the Expected Probe

**Figure 3.**
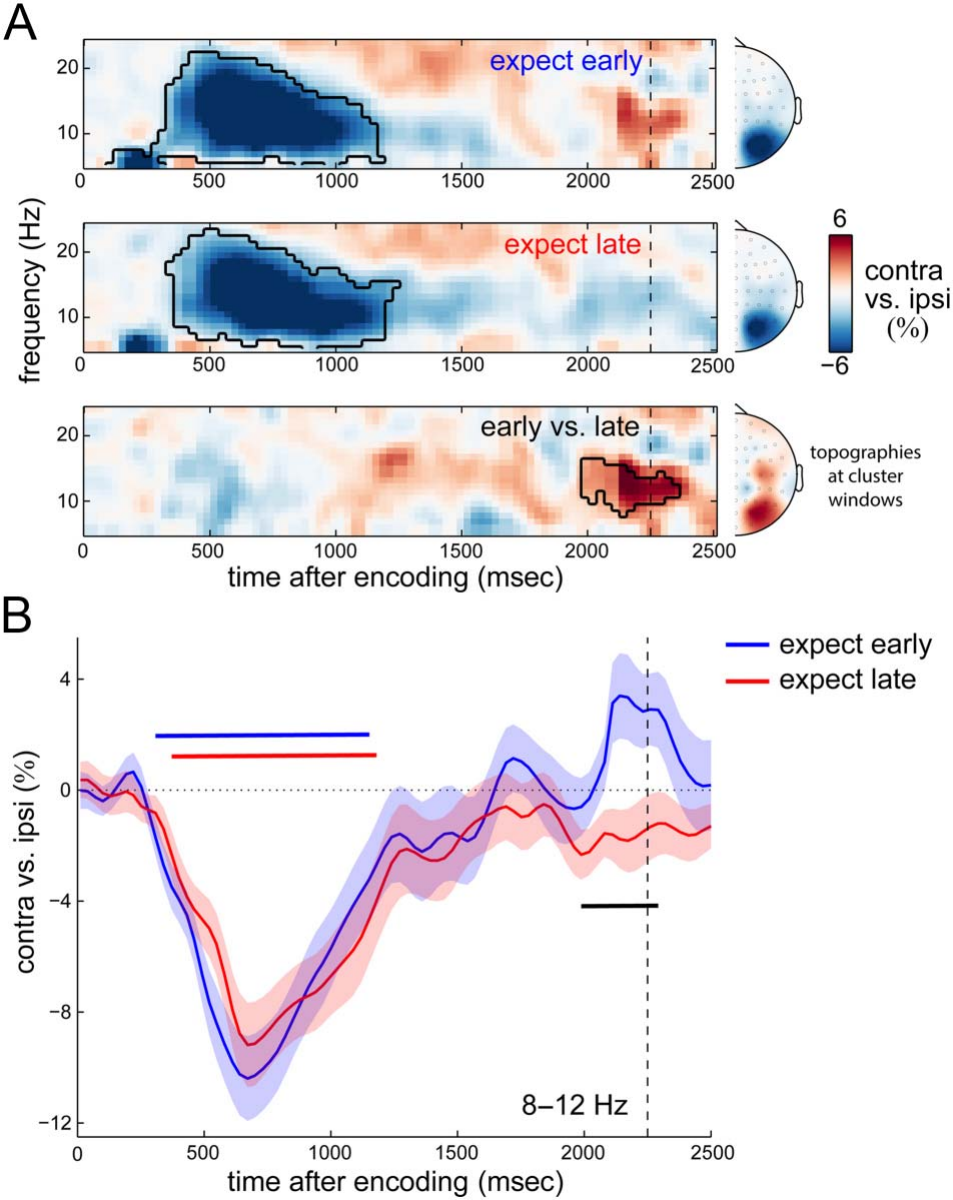
Temporal expectation is associated with a temporally specific spatial reorganization of alpha activity the time of expected memory utilization. (A) Time–frequency maps of neural lateralization (contra- vs. ipsilateral to the location of the relevant memory shapes at encoding) in selected posterior electrodes (see Methods section), separately for expect-early (top) and expect-late (middle) conditions, as well as their difference (bottom). Black outlines indicate significant clusters. Topographies show lateralization collapsed over the time–frequency points of the respective clusters. (B) Time courses of lateralization in the predefined 8- to 12-Hz alpha band. Horizontal lines indicate significant clusters (black for the difference between temporal expectation conditions). Dashed vertical lines indicate the time at which an early probe could occur.

We first focused on alpha lateralization. Figure 3A shows the time- and frequency-resolved lateralization of neural activity in selected posterior electrodes (see Methods section for details on our independent electrode selection), relative to the location of the relevant memory array. In line with prior studies (e.g., Hakim et al., 2019; Lozano-Soldevilla et al., 2014; van Dijk et al., 2010; Sauseng et al., 2009), we observed robust lateralization of alpha power over the posterior scalp reflecting a relative attenuation in contralateral (vs. ipsilateral) electrodes. This lateralization was particularly pronounced in the first second of the memory delay and was highly similar in expect-early and expect-late blocks (Figure 3A, top and middle; cluster *p*_early_ < .0001; cluster *p*_late_ < .0001). Later in the memory delay, however, the pattern of alpha lateralization appeared to depend on temporal expectation. Alpha lateralization in expect-early blocks reversed sign around the time of expected memory use, whereas it remained unchanged in the expect-late blocks. A direct comparison between expect-early and expect-late blocks (Figure 3A, bottom) revealed a significant difference in spectral lateralization (cluster *p* = .017) centered on alpha band activity at the anticipated time of the probe in expect-early blocks. This difference in lateralization also has a posterior topography. Figure 3B shows the associated time courses of 8- to 12-Hz alpha lateralization in expect-early and expect-late blocks and confirms the temporally tuned spatial reorganization of alpha activity by temporal expectation (cluster *p*_early_ = .0002, cluster *p*_late_ = .0002, cluster *p*_early vs. late_ = .0095).

We note that this difference in alpha lateralization between expect-early and expect-late blocks cannot be explained by differences in visual input (probe onset). Time windows for analysis were adjusted to remove any data points that could be contaminated by probe onset (see Methods section for details). Because the clusters of the modulation of alpha lateralization started at 1990 msec, this effect was also unlikely to result from our correction procedure that was applied from 2100 msec onward. Moreover, although our correction procedure resulted in an imbalance of usable expect-early and expect-late trials (from 2100 msec onward), the mean lateralization values that we compared between conditions should not be systematically biased toward lower or higher values by the number of available trials.

### No Effect of Temporal Expectation on the CDA

**Figure 4.**
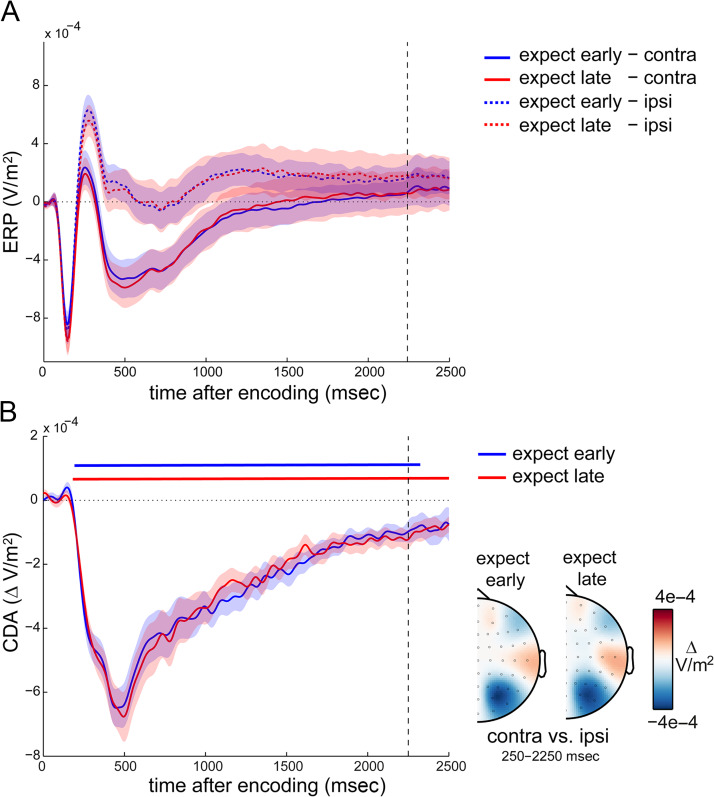
No effect of temporal expectation on the CDA. (A) Event-related potentials in selected posterior electrodes contra- and ipsilateral to the side of the relevant memory items at encoding. (B) CDA waveforms (contra- vs. ipsilateral ERPs in (A) as a function of temporal expectation, together with the associated topographies). Dashed vertical lines indicate the time at which an early probe could occur.

In a similar spirit to the analysis of alpha lateralization, we also investigated the effects of temporal expectation on a second lateralized measure implicated in visual working memory retention after lateralized encoding displays: the CDA (e.g., Hakim et al., 2019; Schmidt & Zelinsky, 2017; Luria et al., 2016; Carlisle et al., 2011; van Dijk et al., 2010; Vogel et al., 2005; Vogel & Machizawa, 2004). Figure 4A shows the ERPs in contra- and ipsilateral posterior EEG electrodes separately for expect-early and expect-late blocks, whereas Figure 4B depicts the associated contra-minus-ipsi difference waves that constitute the CDA. Although, for simplicity, we refer to any lateralized posterior ERP activity as the CDA, we note that the early parts of this component are likely to reflect, or receive contributions from, the N2pc (Luck & Hillyard, 1994) and/or related components (e.g., Töllner, Müller, & Zehetleitner, 2012; Robitaille & Jolicoeur, 2006).

As with the initial alpha lateralization, in both block types, we observed a clear difference between contra- and ipsilateral traces (cluster *p*_early_ < .0001, cluster *p*_late_ < .0001) that were each also characterized by a posterior topography (Figure 4B). However, unlike the alpha lateralization, no reversal in the CDA was observed around the time of expected memory use in the expect-early blocks, and no difference was observed when directly comparing the CDA in expect-early and expect-late blocks (no clusters found; all univariate *p* before multiple-comparison correction ≥ .0774).

## DISCUSSION

The current data provide a clear demonstration that temporal expectations are utilized during visual working memory retention, with consequences for the accuracy and speed of the upcoming memory-guided behavior. The demonstration of a CNV as well as the modulation in alpha lateralization—that were each sensitive to the time of expected memory utilization—provided direct evidence that temporal expectations were used during the memory delay. These demonstrations advance the literatures on both working memory and on temporal expectations in relevant ways.

Regarding working memory, we show that time plays a key role in preparing for a memory-guided behavior. In understanding how working memory bridges past to future, it is thus relevant to consider not only what items (McNab & Klingberg, 2008; Vogel et al., 2005; Griffin & Nobre, 2003) and which features (Niklaus, Nobre, & van Ede, 2017; Lee et al., 2013; Serences et al., 2009) to retain and prioritize in working memory. It is also relevant to consider when our memories will become relevant for guiding a prospective behavior (see also Zokaei et al., 2019; de Vries, van Driel, & Olivers, 2017; Olmos-Solis et al., 2017; van Ede et al., 2017; Wasserman, Grosch, & Nevin, 1982; Perkins, Lydersen, & Beaman, 1973). Working-memory-guided behavior was impaired when items were unexpectedly probed early, but recovered when probed late. Impaired performance could therefore not be because of a weaker memory trace per se (if anything, a weaker trace would be expected after the late interval, because more time would have passed since encoding). Rather, when unexpectedly probed early, participants appeared not to be as ready to use their memories to guide performance. Temporal expectations may thus play a key role in shaping the readiness to utilize the contents of working memory for guiding behavior—possibly mediated by reconfiguring the “coding format” of memory content in anticipation of its expected use (de Vries et al., 2020; Nobre & Stokes, 2019; Christophel, Iamshchinina, Yan, Allefeld, & Haynes, 2018; van Loon, Olmos-Solis, Fahrenfort, & Olivers, 2018; Myers et al., 2017; Lewis-Peacock, Drysdale, Oberauer, & Postle, 2012; Olivers et al., 2011). Notably, whereas prior studies on “memory readiness” all focused on the prioritization of individual memory items, in our case, this dynamic change in readiness applied to the utilization of multiple items in memory collectively.

Regarding temporal expectation, our data make clear that the benefits of temporal expectations are not limited to the type of “pure” perception and action tasks in which they are traditionally investigated: during “blank intervals” while awaiting a sensory stimulus to detect, identify, or discriminate, or awaiting a go-signal to execute a simple motor act (such as a speeded button press). Here, we have investigated temporal expectations while participants retained multiple items in working memory. Prior studies have shown that temporal orienting effects—of the type studied here—can be abolished in the face of concurrent working memory demands (Capizzi, Correa, & Sanabria, 2013; Capizzi, Sanabria, & Correa, 2012). In these dual-task studies, however, temporal expectations did not regard the working memory task itself. Rather, working memory demands served to increase “cognitive load” for the primary temporal expectation task. When temporal expectations do concern the working memory task and predict the time of upcoming working memory utilization, temporal expectations can have profound effects on working-memory-guided behavior, as our data make clear. By showing that temporal expectations can influence the *accuracy* of working-memory-guided behavior, our data further suggest that this influence reflects more than a mere change in response readiness.

### Relation to Other Recent Studies on Temporal Expectations in Working Memory

Our findings build on recent demonstrations for a role of temporal expectations in working memory. For example, temporal expectations regarding the time of stimulus presentation can facilitate the encoding of sensory information into working memory (Wilsch et al., 2015, 2018). In contrast, in the current work, temporal expectations were manifested during memory retention, showing that temporal expectations can also facilitate how information that is already in working memory guides behavior.

In other recent studies from our own laboratory (Zokaei et al., 2019; van Ede et al., 2017), we manipulated temporal expectations to investigate the flexibility of prioritization among memory contents (building on, e.g., de Vries et al., 2020; Rerko & Oberauer, 2013; Lewis-Peacock et al., 2012; Griffin & Nobre, 2003; Oberauer, 2002). In these tasks, probes were equally likely to occur early or late, and we instead varied which memory item was most likely to be probed at each probe time. We now show that temporal expectations can also influence memory-guided behavior in contexts where multiple items in memory collectively become relevant for guiding behavior. The current task further allowed us to investigate the CNV and CDA, two EEG signatures that were not studied in our prior work, which focused instead on alpha lateralization (van Ede et al., 2017) and pupil size (Zokaei et al., 2019).

Another study manipulated the time at which visual templates in working memory would become relevant for guiding visual search and measured micro-saccadic biases to template-matching distractors before the search display (Olmos-Solis et al., 2017). Biases depended on temporal expectation, consistent with bringing the memory template into an active state at the time at which it was expected to guide search (see also de Vries, van Driel, Karacaoglu, & Olivers, 2018; van Loon, Olmos-Solis, & Olivers, 2017; Myers, Rohenkohl, et al., 2015). This is akin to holding all memory content ready for the delayed change detection in the current task.

Complementing the abovementioned studies, here, we investigated temporal expectations in the context of a lateralized change detection task that has been widely used to study visual working memory and its electrophysiological correlates (e.g., Hakim et al., 2019; Fukuda, Kang, & Woodman, 2016; Luria et al., 2016; Vogel et al., 2005; Vogel & Machizawa, 2004). To our knowledge, expected probe time has not previously been investigated in the context of this popular task. We were able to replicate the lateralized alpha and CDA electrophysiological markers associated with this change detection task, putting us in the position to ask whether and how these signatures are also modulated by temporal expectation.

### Alpha Lateralization and CDA Are Differentially Sensitive to Temporal Expectation

One of the more intriguing findings of the current study was the differential sensitivity of alpha lateralization versus the CDA to temporal expectation. Although alpha lateralization underwent significant reorganization in anticipation of the probe array, the CDA appeared unaffected. Thus, the two markers likely capture different information during working memory retention (for related findings, see also Hakim et al., 2019; Fukuda et al., 2016; Myers, Walther, et al., 2015).

What might alpha lateralization reflect in our task? Relevant memory items were lateralized in the encoding display but were always probed centrally. Any differences in lateralization must therefore be attributed to differences in spatial attention and/or retention of the relevant memory items. We propose that alpha likely reflects shifting of spatial attention in our task—first to the relevant side of the display during encoding and early maintenance (regardless of temporal expectation), followed by a spatial shift to the center of the screen in anticipation of the central probe (contingent on temporal expectation). We further speculate that an overshoot in this spatial shift—possibly reflecting a physiological “rebound” from the state that preceded this shift—may account for the apparent “reversal” of lateralization. We note, however, that the reversal in the expect-early condition was not significant by itself but instead contributed to the significant difference in alpha lateralization between expect-early and expect-late conditions. This particular effect thus has to be interpreted with some caution. Moreover, whether this shift just before the time of the expected probe includes a concomitant spatial transformation of the contents of memory (Woodman, Vogel, & Luck, 2012) or a pure attention shift in anticipation of the central task demands remains an interesting question to address in future research.

The CDA, in contrast, showed no modulation by temporal expectation, thus providing further evidence against the earlier suggestion that these two signatures reflect a single underlying neural process (van Dijk et al., 2010) and for its diminished sensitivity to changes in the focus of spatial attention (Hakim et al., 2019; Fukuda et al., 2016). What, then, did the CDA reflect in our task? In our data, the CDA gradually dissipated with time (regardless of temporal expectations). On the basis of these data, we speculate that the CDA reflects a lingering trace resulting from the spatially biased encoding, which then dissipates gradually; at least in cases where it is known in advance that the memory contents will not be probed at the same spatial location where they were encoded. Whether the CDA would have remained sustained and/or been sensitive to temporal expectation if items were probed items at their encoded locations (thereby not requiring any spatial transformation of the encoded memory content) remain relevant questions for future studies.

### Conclusion

The current work provides the clear demonstration of the utility of temporal expectations for guiding upcoming working-memory-guided behavior, as reflected in behavioral performance and the CNV—extending the literatures on both working memory and on temporal expectations in relevant ways. This helps place time on the map as an important, yet underexplored, variable in working memory tasks. As a bonus, we have also presented new findings regarding two key electrophysiological signatures associated with visual working memory in tasks with lateralized displays. We hope our findings will motivate relevant new experimental manipulations that will help further elucidate the role of these signatures in working memory—taking into account both the space and the time of prospective memory use.

## Author Contributions

Wen Jin: Conceptualization; Data curation; Formal analysis; Funding acquisition; Investigation; Methodology, Project administration; Resources; Software; Supervision; Validation; Visualization; Writing - Original Draft; Writing - Review & Editing. Anna C. Nobre: Conceptualization; Data curation; Formal analysis; Funding acquisition; Investigation; Methodology; Project administration; Resources; Software; Supervision; Validation; Visualization; Writing - Original Draft, Writing - Review & Editing. Freek van Ede: Conceptualization; Data curation; Formal analysis; Funding acquisition; Investigation; Methodology; Project administration; Resources; Software; Supervision; Validation; Visualization; Writing - Original Draft, Writing - Review & Editing.
